# A Novel Variant of the PIK3R1 Gene Mutation Associated With SHORT Syndrome and Agammaglobulinemia

**DOI:** 10.7759/cureus.62983

**Published:** 2024-06-23

**Authors:** Juwairiya Syed Iqbaluddin, Funda Cipe, Suleiman Al-Hammadi, Sinan Yavuz, Nader Francis, Amal Sherif

**Affiliations:** 1 Pediatrics, Al Qassimi Women's and Children's Hospital, Sharjah, ARE; 2 Pediatric Allergy and Immunology, Al Qassimi Women's and Children's Hospital, Sharjah, ARE; 3 Pediatric Allergy and Immunology, Mohammed Bin Rashid University, Al Jalila Children's Specialty Hospital, Dubai, ARE; 4 Pediatric Pulmonology, Al Qassimi Women's and Children's Hospital, Sharjah, ARE; 5 General Pediatrics, Al Qassimi Women's and Children's Hospital, Sharjah, ARE

**Keywords:** pik3r1 gene, intravenous immunoglobulin (ivig), immunodeficiencies, agammaglobulinemia, short syndrome

## Abstract

Primary immunodeficiencies are disorders of the immune system often caused by mutations of genes required for lymphocyte development. Phosphoinositide-3-kinase regulatory subunit 1 (PIK3R1) gene mutations are associated with SHORT syndrome, a rare multisystem disease. The name stands for Short stature, Hyperextensibility, Ocular depression, Rieger anomaly and Teething delay. Our case describes a child who presented with agammaglobulinemia with phenotypical features of SHORT syndrome. Upon further investigation, he was found to have a rare variant of the PIK3R1 gene mutation. This new mutation combines two distinct diseases with the same gene defect, which otherwise has been reported as two separate entities. The objective of this report is to identify a new gene mutation associated with SHORT syndrome along with agammaglobulinemia and to highlight the importance of recognizing the features of SHORT syndrome. We describe a nine-year-old male who presented with developmental delay and recurrent infections at the age of 12 months. Immunological evaluation revealed agammaglobulinemia and he has been scheduled for regular intravenous immunoglobulin replacement therapy. In view of characteristic syndromic physical features, speech and teething delay, we investigated further for the underlying genetic reason for agammaglobulinemia. The molecular analysis demonstrated a rare homozygous variant, c.244dup, in the PIK3R1 gene. This case reveals the association of the PIK3R1 gene mutation with agammaglobulinemia and SHORT syndrome. It further demonstrates the discovery of a new pathological variant of the gene. A detailed history and examination along with an immunological and genetic workup should be carried out for children with certain distinct phenotypical features. SHORT syndrome has specific characteristics that call for awareness and early recognition for prompt diagnosis and intervention. Emphasis is placed on genetic counseling as the disease is inherited in an autosomal recessive pattern, as demonstrated by molecular genetic studies.

## Introduction

The phosphatidylinositol 3-kinase (PI3K)-AKT-mTOR pathway is a major intracellular network that leads to cell proliferation. Each class of PI3K is made up of one p110 catalytic subunit (p110α/PIK3CA, p110β/PIK3CB or p110δ/PIK3CD) and one p85 regulatory subunit (p85α, p55α, p50α, p85β or p55γ). The p85 subunit consists of two closely related proteins, p85α (phosphoinositide-3-kinase regulatory subunit 1 (PIK3R1)) and p85β (PIK3R2) [1]. One of the main functions of PIK3R1 is to inhibit PI3K signaling [1].

Heterozygous PIK3R1 mutations are the major cause of SHORT syndrome (each letter in the term SHORT represents a feature: (S) Short stature, (H) Hyperextensibility of joints and/or Hernia, (O) Ocular depression, (R) Rieger anomaly, (T) Teething delay). The mutation suggests that the molecular mechanism of the disease might involve downregulation of the PI3K-AKT-mTOR pathway [2]. In addition, homozygous mutations in the PIK3R1 gene resulting in the absence of p85α have been defined as a cause of agammaglobulinemia [3,4]. Besides this, autosomal dominant loss-of-function mutations in the PIK3R1 gene encoding the p85α, p55α and p50α regulatory subunits cause activated PI3-kinase-δ syndrome type 2 (APDS2) [5].

The designation SHORT syndrome was coined by Gorlin et al. [6] to reflect several of the most striking clinical features of the syndrome. The proportion of individuals with SHORT syndrome caused by a de novo pathogenic variant is unknown but appears to be significant. Each child of an individual with the SHORT syndrome has a 50% chance of inheriting the pathogenic variant [7].

We describe a nine-year-old male who initially presented with developmental and speech delays, recurrent infections and skin lesions at 12 months of age. Immunological evaluation revealed agammaglobulinemia, and he was placed on regular intravenous immunoglobulin (IVIG) replacement therapy. During follow-up, due to his syndromic physical features, speech delays, and delayed teething, we investigated the underlying genetic cause of his agammaglobulinemia. Molecular analysis revealed a rare, novel homozygous variant c.244dup in the PIK3R1 gene. Mutations in this gene have been associated with both SHORT syndrome and autosomal recessive agammaglobulinemia as separate clinical entities. Our patient exhibits clinical and laboratory findings consistent with both SHORT syndrome and agammaglobulinemia due to this novel mutation.

## Case presentation

A nine-year-old boy was born as an extreme preterm at 26 weeks of gestation to an unbooked primigravida mother. Birth weight was 1038 grams while length was 38 cm which were appropriate for the gestational age. The baby was intubated and shifted to the neonatal intensive care unit immediately after birth due to respiratory distress syndrome. He had three months of comprehensive stay in the hospital.

Family history revealed parents were first-degree relatives and there was no similar history in the extended family. The child has had multiple visits to the emergency due to recurrent upper and lower tract infections after six months of age. He later presented with vomiting and diarrhea and was admitted as a case of gastroenteritis with febrile neutropenia. At the age of 12 months, the child was brought to the hospital due to scrotal swelling, multiple ulcers were observed with slough and erythema, and he was treated with antibiotics however skin lesions recurred several times later on. Skin revealed multiple skin papules and furuncles. In regards to his skin lesions, he underwent debridement along with a skin biopsy which showed acute macro-inflammatory infiltrate in the dermis, parakeratosis, intraepidermal clefts, and intraepidermal neutrophils, suggestive of the non-specific acute necro-inflammatory lesion (skin biopsy image not available). Throughout this extended admission, he underwent comprehensive evaluations for primary immunodeficiencies, which included assessments of basic immunoglobulin levels and lymphocyte subsets. Agammaglobulinemia was established with absent peripheral B cells (CD19: 0.06%), therefore X-linked agammaglobulinemia (XLA) was regarded as the most probable diagnosis (Table 1). 

**Table 1:** Immunological laboratory evaluation of the patient

Immunoglobulin (Ig) level	At time of diagnosis (2013; age 15 months)	Latest (2023; age 9 years)	Normal range
IgA (mg/dL)	< 1	<6.6	27-195
IgG (mg/dL)	11	559	572-1474
IgM (mg/dL)	1.2	<4.5	24-201
IgE (IU/mL)	< 1.5	<1.5	0-90
Lymphocyte subsets			
Absolute lymphocyte count /(µL)	3310	2810	1500-4500
CD3 %	98	95	55-82
CD4 %	85	63	27-57
CD8 %	7.8	20	14-34
CD19 %	0.2	0.06	9-29
CD16+56 %	0.1	3	3-12

Upon acquiring further history, the mother informed us that the child experienced delays in reaching appropriate developmental milestones while growing up. Additionally, he had hearing impairment, speech delay and delayed dentition. At the age of nine years, his weight was 22 kg (-1.92 SD, <third percentile) and his height was 131 cm (0.40 SD, 25th percentile). Physical examination revealed a child with a triangular face, deep-set eyes, prominent ears, and notably broad and sparse eyebrows. He exhibited clinodactyly, hyperextensible joints and lipodystrophy on his cheeks (consent for a facial photograph was denied). 

Four years after his diagnosis of XLA, genetic testing was considered due to his increasingly apparent syndromic physical features. Whole exome sequencing was performed, revealing a novel pathogenic homozygous frameshift mutation NM_181523.3:c.244dup(p.(lle82Asnfs*24) chr5:67522740) in the PIK3R1 gene. This mutation causes a premature stop codon and was identified without any other heterozygous variant, suggesting it contributes to the clinical presentation. In light of the clinical findings suggestive of SHORT syndrome, the patient underwent further examination. Ophthalmological examination revealed no abnormalities such as Rieger anomaly; he only had hypermetropia with astigmatism and was prescribed eyeglasses. Hearing assessment and other ear, nose, and throat examinations were normal.

He has been receiving regular IVIG replacement therapy every three to four weeks for the past eight years. With this preventive IVIG treatment, the frequency of his infections has decreased to levels comparable to those observed in healthy children. He is also on prophylactic azithromycin. If he develops infections, a plan is in place for aggressive treatment with antibiotics, antifungals, or antivirals. He requires laboratory evaluation including complete blood count, liver function tests, renal function tests and immunoglobulin levels either as indicated or regularly every three months. To monitor for complications of the disease, a multidisciplinary team involvement is planned for early detection and management.

## Discussion

PI3Ks are broadly expressed group of enzymes that plays a crucial roles in various cellular activities, including proliferation, migration, metabolism, survival, growth, and apoptosis. In addition, this gene regulates the activity of hormones, growth factors, and metabolic processes [8-10].

SHORT syndrome is a rare genetic disorder characterized by the presence of multiple congenital abnormalities affecting internal organs [8,9]. Due to the limited number of affected individuals and the diverse clinical manifestations associated with SHORT syndrome, pinpointing the specific mutation responsible for this disorder can pose challenges [9]. SHORT syndrome is attributed to heterozygous mutations of PIK3R1 located on chromosome 5. The exact proportion of cases resulting from de novo mutations is currently unknown [8]. Our case showed a homozygous mutation in the PIK3R1 gene and revealed some features of SHORT syndrome such as hyperextensibility, vision abnormalities, lack of fat tissue, triangular face, extroverted ears, ocular depression, developmental and teething delay. Because this syndrome is inherited autosomal dominant, parents can be expected to have some syndromic features. In our case, the patient’s parents did not have any finding of SHORT syndrome.

A specific set of clinical criteria for the diagnosis of SHORT syndrome has not been established yet however the disease can be suspected based on the typical physical features and concomitant congenital anomalies. Molecular analysis remains essential for confirming the diagnosis of this syndrome [8,9]. Among all ‘SHORT’ initials, only H, O, and T were present in our patient. Dyment et al. noted that the abbreviated features associated with SHORT syndrome do not encompass the complete spectrum of the clinical phenotype. This includes distinctive facial characteristics such as a triangular facial shape, absence of facial fat, and underdeveloped nasal wings with a protruding columella. Additionally, nearly all individuals with SHORT syndrome experience partial lipodystrophy, insulin resistance, nephrocalcinosis and hearing impairments. It is worth mentioning that individuals with this syndrome typically achieve normal developmental milestones and exhibit normal cognitive abilities [2]. Furthermore, Reardon and Temple reported a previously undocumented feature of SHORT syndrome, suggesting that calcium metabolism may also be affected [11].

In one case series in which clinical features of 32 individuals were reviewed with genetically confirmed SHORT syndrome, the major features described in the SHORT acronym were not universally seen and only half (52%) had four or more of the classic features. The most common features included intrauterine and postnatal growth restriction, lipoatrophy, and a characteristic facial gestalt characterized by a progeroid appearance with prominent forehead, triangular face, deep-set eyes, hypoplastic alae nasi, midface hypoplasia, small chin, low-set ears, and downturned mouth. Out of the 29 cases examined, only 10 presented with hyperextensible joints or inguinal hernia, while Rieger anomaly was found in just 13 out of 30 cases, although some patients exhibited other anterior chamber defects. Insulin resistance was observed in 13 out of 17 patients and diabetes was present in nine out of 14 individuals. Cardiac anomalies were detected in three patients, while sensorineural deafness was found in five patients. Despite our patient not exhibiting any signs of insulin resistance, diabetes or metabolic abnormalities at present, it is important to monitor them for the following symptoms [12].

When it comes to primary immunodeficiencies, APDS2, hyper IgM-like syndrome and agammaglobulinemia have been identified as related to the same gene mutations so far. The first report was in 2012, when Conley et al. identified a homozygous truncating variant in the PIK3R1 gene causing autosomal recessive agammaglobulinemia [3]. The second case report of a homozygous mutation within the PIK3R1 gene leading to agammaglobulinemia was defined in 2018 by Tang et al. [4]. The gene that encodes PIK3R1 (p85α) also encodes two additional regulatory isoforms, p55α and p50α. It has been shown that mice, which are null for p85α but retain expression of p55α and p50α, are viable but have reduced numbers of B cells with elevated numbers of pro-B cells like XLA [3,13]. While mouse studies have shown that loss of p85α results in increased sensitivity to insulin, defective platelet function, abnormal mast cell development, and increased production of IL-12 by dendritic cells, in the previous agammaglobulinemia patients, clinical consequences of the p85α defect have been relatively B cell-specific [3,13]. In our case, the homozygous mutation causing a premature stop codon in the PIK3R1 gene can explain agammaglobulinemia clinical findings although we could not analyze p85α, p50α and p55α protein expressions in our patient because of unavailability of techniques. We can only speculate that this new frameshift mutation would lead to a stop codon in the p85α transcript which results in nonsense-mediated decay and very low expression of p85α, however p50α and p55α isoforms would be left unaffected. The location of this mutation might contribute to the clinical dysmorphic features observed, as no dysmorphism was described in the two previously reported cases, unlike our patient.

Following the identification of agammaglobulinemia associated with PIK3R1 gene mutations in four patients from three unrelated families with autosomal dominant immunodeficiency and lymphoproliferation, heterozygous mutations of the PIK3R1 gene were discovered. These monoallelic loss-of-function (LOF) mutations in the PIK3R1 gene caused APDS2. APDS2 is defined as a primary antibody deficiency, developmental abnormalities within the B and T lymph cell compartments and immune dysregulation [14,15]. It has been shown that the genetic defect in APDS is shared with that found in SHORT syndrome, as evidenced by a similar case of ADPS type 2 [16].

In this case report, while the clinical and laboratory findings indicated agammaglobulinemia, the phenotypical features of the patient prompted suspicion of one of the syndromic primary immunodeficiencies, prompting us to conduct genetic testing. To our knowledge, our case is the first case describing agammaglobulinemia together with SHORT syndrome atypical features. This novel pathological homozygous mutation in the PIK3R1 gene highlights the association of agammaglobulinemia with SHORT syndrome.

In comparison to other reported cases, we can speculate that the type of genetic mutation and its location on the gene may play an important role in determining the clinical presentation of the syndrome. It can be suggested that all patients with PIK3R1 mutation should be evaluated for the clinical signs of SHORT syndrome as well and vice versa [17].

## Conclusions

This case reveals the association of the PIK3R1 gene mutation with agammaglobulinemia and SHORT syndrome. It furthermore demonstrates the discovery of a new pathological variant of the gene. A detailed history and examination, along with immunological and genetic workup, should be carried out for children with certain distinct phenotypical features. SHORT syndrome has specific characteristics that call for awareness and early recognition for prompt diagnosis and intervention. Emphasis should be made on genetic counseling as the disease may be inherited in an autosomal recessive pattern as demonstrated by molecular genetic studies.
